# Large mediastinal mass diagnosed as Nocardia infection by endobronchial ultrasound-guided transbronchial needle aspiration in a ceramic worker: A case report

**DOI:** 10.3389/fsurg.2022.983074

**Published:** 2023-01-06

**Authors:** Xiaoshan Su, Lin Chen, Zesen Zhuang, Yixiang Zhang, Xiaoping Lin, Jiaming Huang, Zhixing Zhu, Huaping Zhang, Weijing Wu

**Affiliations:** ^1^ Department of Pulmonary and Critical Care Medicine, The Second Affiliated Hospital of Fujian Medical University, Respirology Medicine Centre of Fujian Province, Quanzhou, China; ^2^ Department of Medical Imaging, Quanzhou Jinjiang Anhai Hospital, Quanzhou, China; ^3^ Department of Laboratory Medicine, The Second Affiliated Hospital of Fujian Medical University, Quanzhou, China

**Keywords:** nocardia, mediastinal mass, EBUS-TBNA, weakly acid-fast stain, MNGs

## Abstract

**Background:**

Nocardia is a ubiquitous soil saprophyte transmitted through airborne or direct cutaneous inoculation routes. Although Nocardia is more common in immunocompromised patients, Nocardia may also arise in apparently immunocompetent patients.

**Case presentation:**

We report a rare case of Nocardia infection presenting as a large mediastinal mass in an immunocompetent ceramic worker. A 54-year-old man with no previous history of immune dysfunction, a ceramic worker by profession, was referred and admitted to our hospital because of a persistent fever for 19 days. Chest CT showed a large middle mediastinal mass. However, conventional anti-infective treatment was ineffective. Under the guidance of the Virtual bronchoscopic navigation (VBN) system, he underwent Endobronchial ultrasound-guided transbronchial needle aspiration (EBUS-TBNA). The purulent exudate obtained by EBUS-TBNA was further identified as Nocardia by weak acid-fast and metagenomic next-generation sequencing (mNGS). He was subsequently treated with intravenous imipenem/amikacin, switched to intravenous imipenem and oral trimethoprim/sulfamethoxazole, and the clinical symptoms were significantly improved.

**Conclusions:**

Even in immunocompetent patients, Nocardiosis cannot be excluded. For the public, especially soil contact workers, precautions should be taken to avoid Nocardia infection from occupational exposure. This rare case may provide a diagnosis and treatment reference for clinicians.

## Background

The Nocardia genus is a Gram-positive, branching, filamentous bacterium that is ubiquitous in soil and is transmitted by airborne or direct skin inoculation routes. Nocardiosis is an opportunistic infection that often occurs in immunocompromised patients, such as those with acquired immune deficiency syndrome (AIDS), and rarely in patients with normal immune function. The specific site of Nocardia infection is the respiratory tract, with subsequent spread to distant organs. Nocardia infection could commonly manifest in the pulmonary, central nervous, and cutaneous systems (1, 2). Diagnosis of pulmonary Nocardia is challenging due to Pulmonary Nocardia being a rare condition with variable and non-specific clinical presentation. Nocardia can have high morbidity and mortality, especially in patients with immunocompromised or comorbidities (3, 4). A timely and accurate diagnosis of pulmonary Nocardia is a challenging and critical task for further effective treatment. Here, we report a rare case of a large mediastinal mass caused by Nocardia in an immunocompetent patient and describe the clinical and epidemiological findings and timely diagnosis and management.

## Case presentation

**Figure 1:**
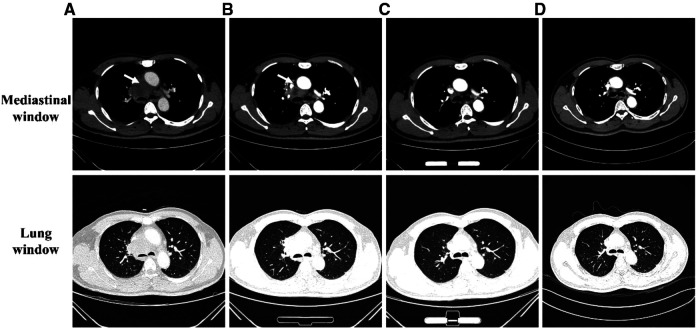
The dynamic changes in enhanced chest CT images at different time points. Mediastinal (top) and lung window (bottom). (**A**) At admission, Chest CT showed a mediastinal mass measuring 7.76 × 4.55 cm (arrow). (**B**) During discharge, Chest CT showed a decrease in the size of mediastinal mass to 5.53 × 4.51 cm (arrow). (**C**) After six months of treatment, Chest CT showed obvious absorbance of the mediastinal lesions. (**D**) One and a half years follow-up Chest CT showed obvious absorbance of the mediastinal lesions.

A 54-year-old male, a ceramic worker with no previous history of immune dysfunction, was admitted to the hospital with persistent fever for 19 days. The patient had a cough occasionally and joint soreness but denied any other symptoms. On initial clinical evaluation, the patient's body temperature was 38.4 °C, and other signs were within normal limits. Pulmonary, abdominal, cardiac, and neurologic examinations showed unremarkable findings. Laboratory tests revealed that the patient had leukocytosis (20.72 × 10^9^/ml) with 87.4% neutrophils. His C-reactive protein level was high (90.61 mg/L), and a procalcitonin level was slightly increased (0.173 ng/ml). Contrast-enhanced chest CT revealed a large mediastinal mass measuring approximately 7.76 × 4.55 cm (Figure 1A). The rest of the examination was regular.

**Figure 2:**
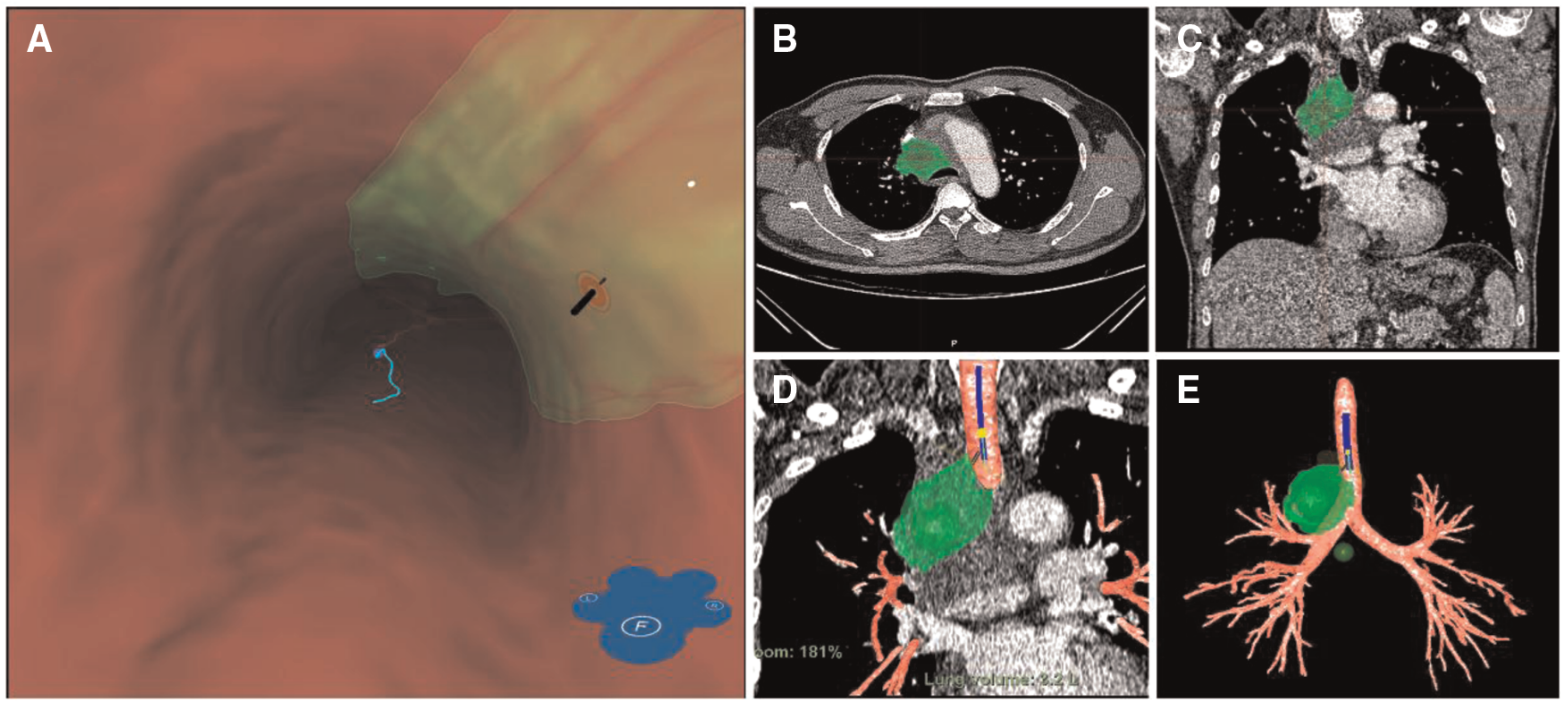
Three-dimensional anatomical structures were reconstructed using a VBN system (archimedes virtual bronchoscopy navigation system). (**A**) Intraluminal view and puncture sight (green). (**B**) Axial view of the location of the mediastinal lesion (green dot). (**C**) Coronal view of the location of the mediastinal lesion (green dot). (**D**) VBN image showed the target lesion (green dot) and the route for TBNA (light blue line). (**E**) the anatomical structure of the mediastinal mass and bronchial tree.

**Figure 3:**
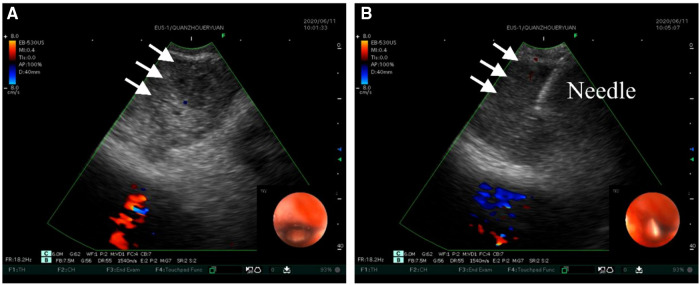
Ultrasound images of EBUS-TBNA. (**A**) Convex-probe ultrasound demonstrates the hypoechoic lesion (arrows), while the endoscopic Doppler image reflects the blood flow within the right atrium. (**B**) EBUS-TBNA was performed using a 22-gauge needle.

**Figure 4:**
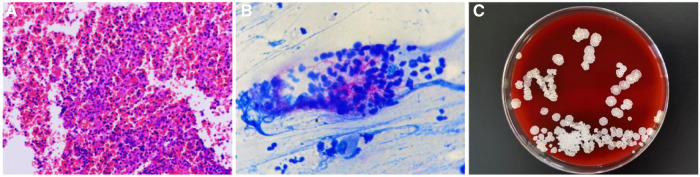
(**A**) H&E stain for the biopsy showed more purulent secretions and a few lymphocytes and macrophages (X40). (**B**) Kinyoun stain for the samples on the aspirate smear showed numerous weakly acid-fast and branching filamentous rod bacteria (X100). (**C**) The biopsy samples were incubated at 35 °C for 7 days and formed milky colonies floating on the medium liquid surface. The medium liquid was clarified.

At initial admission, the patient was treated with empiric antibiotic therapy, including piperacillin and sulbactam. However, he still had a recurrent fever, and there was no significant improvement in inflammatory indicators and blood routine examination. To determine the etiology, the patient underwent Endobronchial ultrasound-guided transbronchial needle aspiration (EBUS-TBNA) on the fifth day of admission. The Virtual bronchoscopic navigation (VBN) system is a method to guide the bronchoscope to the lesion by making a bronchial path on a virtual image. The digitized information from the patient's CT scan was imported into the Archimedes VBN system, in which multislice views of the chest and virtual bronchoscopy images were reconstructed. The VBN system shows the bronchial tree, the anatomical structure of the mediastinal mass and visualizes the best path to reach the mediastinal mass (Figures 2A–E). With the guidance of the VBN system, the bronchoscope was navigated to the target bronchus and advanced to the lesion, and then EBUS-TBNA was performed to obtain purulent exudate (Figures 3A–D). Biopsy showed more purulent secretions and a few lymphocytes and macrophages (Figure 4A). Microscopic analysis revealed numerous weakly acid-fast and branching filamentous rod bacteria were identified from the samples on the aspirate smear, and a presumptive microbiological diagnosis (Nocardia) was made (Figure 4B). Metagenomic next-generation sequencing (mNGS) subsequently identified the pathogens as Nocardia species (Nocardia arizonensis and Nocardia cyriacigeorgica). And after seven days of culture, the cultures of purulent exudates ultimately grew the Nocardia species (Figure 4C).

After microscopic examination and mNGS confirmed Nocardia, intravenous imipenem/amikacin was given. The clinical symptoms of the patient were significantly improved after 6 days of treatment. Then he was switched to intravenous imipenem with oral trimethoprim/sulfamethoxazole (TMP/SMX, 80 mg of TMP, and 400 mg of SMZ/tablet) 3 tablets q6h. One month after treatment, the patient improved, and their Chest CT showed a decrease in the size of the mediastinal mass (Figure 1B). He was discharged and continued to be treated with oral TMP/SMX 3 tablets q8h and sodium bicarbonate for 3 months. Over the next 3 months, he continued on oral TMP/SMX 2 tablets q8h and sodium bicarbonate. After six months of treatment, the patient was asymptomatic. His CT showed significant improvement in the mediastinal mass size (Figure 1C). At one and a half years of follow-up, the patient recovered well, and no complications were noted (Figure 1D).

## Discussion and conclusions

Nocardia is ubiquitous soil saprophytes transmitted by either airborne or direct cutaneous inoculation routes. Although Nocardia more frequently causes invasive infections in immunocompromised patients, it can also occur in immunocompetent patients. Pulmonary Nocardia could manifest pulmonary airspace consolidation, pulmonary nodules, pulmonary infiltrates, cavitation, and pleural effusion (5). Herein, we report an immunocompetent patient who had a large mediastinal mass and presented with a fever. The patient was a ceramic worker who had frequent exposure to soil and may have acquired Nocardia infection from the soil. For the public, especially soil contact workers, precautions should be taken to avoid Nocardia infection from occupational exposure. In our case, occupation-related Nocardia infection may have been reported for the first time.

Nocardia infection presenting as a mediastinal mass is a rare type, and only 9 cases of this disease have been previously reported (6–12). The clinical, diagnostics and treatment features of 9 cases were summarized in Table 1. Invasive methods may be required to obtain a tissue diagnosis to guide treatment when evaluating mediastinal masses. Endobronchial ultrasound-guided transbronchial needle aspiration (EBUS-TBNA) is an invaluable technique in assessing patients with mediastinal and hilar lesions (13). EBUS-TBNA is mainly used for staging non-small cell lung cancer, for diagnosing lung cancer without endobronchial lesions, and for diagnosing benign (especially tuberculosis and sarcoidosis) and malignant mediastinal lesions (14). Virtual bronchoscopic navigation (VBN) is generally used to guide the diagnosis of peripheral pulmonary lesions (especially peripheral nodules <2 cm). VBN facilitates safe and effective sampling of peripulmonary lesions, independent of bronchial sign location, lesion size, and presence or absence of a bronchus sign (15). Moreover, VBN improved the diagnostic accuracy of mediastinal lesions by visualizing mediastinal lesions and accurately identifying puncture sites (16). The VBN system provides an accurate and virtual map for intra- and extra- bronchial landmarks of hilar and mediastinal lymph nodes, thereby increasing the chances of proper collection and minimizing the risk of major bleeding. This may improve the diagnostic accuracy of mediastinal masses and/or lymphadenopathy and assist bronchoscopists in practicing EBUS-TBNA (16). In this case, we successfully diagnosed a >7 cm mediastinal mass using VBN-guided EBUS-TBNA.

**Table 1:** Case summary of Nocardia infection presenting as a mediastinal mass.

References	Age	Clinical presentation	Lesion location	Lesion size (cm)	Surgical operation	Diagnostic Modality	Treatment	Immunocompromised
Kim 2016 (6)	64	Dyspnea and chest wall pain	Right anterior cardiophrenic angle	4	Video-assisted thoracic surgery	16S rRNA sequencing	TMP-SMX	Immunocompetent
Salazar2013 (7)	30	Cough, hoarseness, and shortness of breath	Mediastinal mass	7 × 5 × 9	Cervical mediastinoscopy and biopsies of mediastinal mass	DNA sequencing and sputum cultures	Imipenem and linezolid	A renal transplant recipient
Jastrzembski2002 (8)	32	Dyspnea, cough, and fever	Large right mediastinal mass	NA	Transverse parasternal thoracotomy	mediastinal fluid cultures	TMP-SMX	Patient with sarcoidosis
Maya 2014 (9)	29	Fever and productive cough	Mid-Mediastinum	NA	A biopsy from the mediastinal mass	Culture and polymerase chain reaction	Imipenem and cotrimoxazole	Immunocompetent
Chaya2006 (10)	60	Productive cough	Mediastinal mass	3.5	EUS−FNA	Romanowsky stain and Gomori methenamine silver−stained	NA	NA
Chaya2006 (10)	26	Nonproductive cough	Mediastinal lymphadenopathy	NA	EUS−FNA	Papanicolaou stain and Kinyoun acid−fast stain	NA	HIV−positive
Chaya2006 (10)	35	Cough	Mediastinal Mass	NA	EUS−FNA	Kinyoun acid−fast stain	NA	HIV−positive
El-Herte2012 (11)	49	Chest pain, fever, chills, sweating, cough, and greenish sputum production	Anterior mediastinal mas	NA	Median sternotomy and biopsy of suspicious tissue	NA	Imipenem, amikacin, and TMP-SMX	Patient with myasthenia gravis
Dawood 2020 (12)	59	Chronic dyspnea, fatigue, and myalgias	Lymphadenopathy in the subcarinal area	NA	Transbronchial needle aspiration	Cytopathologic examination	TMP-SMX and linezolid	Refractory and relapsed acute myeloblastic leukemia

EUS−FNA, Endoscopic ultrasound-guided fine−needle aspiration; TMP-SMX, Trimethoprim/sulfamethoxazole; HIV, human immunodeficiency viru.

Diagnosis of Nocardia is challenging because Nocardia species grow very slowly and are difficult to culture. This could lead to delays in diagnosis and treatment. Acid-fast staining and mNGS can be a fast and definite diagnostic method for Nocardia species. In this case, we successfully treated this rare infection through this rapid diagnosis and aimed to raise Nocardia diagnosis awareness among clinicians.

Subacute to chronic respiratory symptoms, elevated inflammatory markers, a mediastinal mass, a history of soil-related occupational exposure, and the absence of common respiratory pathogens on assessment was high indicators of suspected Nocardia infection. Once Nocardia infection is confirmed, prompt antibiotic therapy should be administered immediately. Sulphonamides are the first-line drugs for treating Nocardia infections, and trimethoprim-sulfamethoxazole (TMP-SMX) is considered the first choice for the treatment of susceptible strains. These appropriate medications can lead to significant radiological improvement.

## Data Availability

The original contributions presented in the study are included in the article/Supplementary Material, further inquiries can be directed to the corresponding author/s.

## References

[1] Rosen LB Rocha Pereira N Figueiredo C Fiske LC Ressner RA Hong JC Nocardia-induced granulocyte macrophage colony-stimulating factor is neutralized by autoantibodies in disseminated/extrapulmonary nocardiosis. Clin Infect Dis. (2015) 60:1017–25. 10.1093/cid/ciu968 25472947 PMC4366584

[2] Soleimani M Masoumi A Khodavaisy S Heidari M Haydar AA Izadi A. Current diagnostic tools and management modalities of Nocardia keratitis. J Ophthalmic Inflamm Infect. (2020) 10:36. 10.1186/s12348-020-00228-w 33263838 PMC7710777

[3] Lynch JP Reid G Clark NM. Nocardia spp.: a rare cause of pneumonia globally. Semin Respir Crit Care Med. (2020) 41:538–54. 10.1055/s-0040-1708816 32629491

[4] Barry M AlShehri S Alguhani A Barry M Alhijji A Binkhamis K A fatal case of disseminated nocardiosis due to Nocardia otitidiscaviarum resistant to trimethoprim-sulfamethoxazole: case report and literature review. Ann Clin Microbiol Antimicrob. (2022) 21:17. 10.1186/s12941-022-00511-9 35578282 PMC9112502

[5] Yadav P Kumar D Meena DS Bohra GK Jain V Garg P Clinical features, radiological findings, and treatment outcomes in patients with pulmonary nocardiosis: a retrospective analysis. Cureus. (2021) 13:e17250. 10.7759/cureus.17250 34540476 PMC8445149

[6] Kim J Kang M Kim J Jung S Park J Lee D A case of Nocardia farcinica pneumonia and mediastinitis in an immunocompetent patient. Tuberc Respir Dis (Seoul). (2016) 79:101–3. 10.4046/trd.2016.79.2.101 27066088 PMC4823182

[7] Salazar MN Wray D Denlinger C Srinivas T Thomas B Posadas A. Mediastinal mass and pericardial tamponade in a renal transplant recipient: a rare case of nocardia infection. Am J Case Rep. (2013) 14:295–9. 10.12659/AJCR.889383 23940824 PMC3738093

[8] Jastrzembski SA Teirstein AS Herman SD DePalo LR Lento PA. Nocardiosis presenting as an anterior mediastinal mass in a patient with sarcoidosis. Mt Sinai J Med. (2002) 69:350–3. 12415330

[9] Abu-Gazala M Engel A Stern A Guralnik L. An unusual case of nocardiosis presented as a mediastinal mass in an immunocompetent patient. Am J Respir Crit Care Med. (2014) 189:492–3. 10.1164/rccm.201308-1546LE 24528321

[10] Chaya CT Schnadig V Gupta P Logrono R Bhutani MS. Endoscopic ultrasound-guided fine-needle aspiration for diagnosis of an infectious mediastinal mass and/or lymphadenopathy. Endoscopy. (2006) 38(Suppl 2):E99–E101. 10.1055/s-2006-944867 17366435

[11] El-Herte RI Kanj SS Araj GF Chami H Gharzuddine W. First report of Nocardia asiatica presenting as an anterior mediastinal mass in a patient with Myasthenia Gravis: a case report and review of the literature. Case Rep Infect Dis. (2012) 2012:325767. 10.1155/2012/325767 22844621 PMC3403161

[12] Dawood W Evans SE Grosu H. Mediastinal lymphadenitis due to Nocardia infection. J Bronchology Interv Pulmonol. (2020) 27:e48–51. 10.1097/LBR.0000000000000658 32569081

[13] Chen C Mu C-Y Su M-Q Mao J-Y Zhu Y-H Huang J-A. Endobronchial ultrasound-guided transbronchial needle aspiration increases the yield of transbronchial lung biopsy for the evaluation of peribronchial lesions. Chin Med J (Engl). (2017) 130:11–4. 10.4103/0366-6999.196567 28051017 PMC5221100

[14] Medford ARL Bennett JA Free CM Agrawal S. Endobronchial ultrasound guided transbronchial needle aspiration. Postgrad Med J. (2010) 86:106–15. 10.1136/pgmj.2009.089391 20145060

[15] Sun J Criner GJ Dibardino D Li S Nader D Lam B Efficacy and safety of virtual bronchoscopic navigation with fused fluoroscopy and vessel mapping for access of pulmonary lesions. Respirology. (2022) 27:357–65. 10.1111/resp.14224 35212090

[16] Lan F Yue Y Shen H Shen H Wang Q Yu X Multi-dimensional display of Wang's lymph node map using virtual bronchoscopic navigation system. Front Mol Biosci. (2021) 8:679442. 10.3389/fmolb.2021.679442 34164434 PMC8215157

